# The ERF transcription factor TaERF13-2B functions as a negative regulator of drought tolerance in *Arabidopsis* and wheat

**DOI:** 10.3389/fpls.2025.1535850

**Published:** 2025-03-27

**Authors:** Yang Yu, Conglei Wang, Jianhe Wang, Qingfen Xu, Shuangxing Zhang, Tianqi Song, Guodong Li, Dan Liang, Gang Feng

**Affiliations:** ^1^ Institute of Crop Sciences, Tianjin Academy of Agricultural Sciences, Tianjin, China; ^2^ Tianjin Crop Research Institute, The Key Laboratory of Crop Genetics and Breeding, Tianjin, China; ^3^ College of Agronomy, Northwest Agricultural and Forestry University, Xianyang, China; ^4^ Agricultural Research Institute, Xilin Gol League Institute of Agricultural and Pastoral Sciences, Xilinhot, China

**Keywords:** wheat, drought, ERF, TaERF13-2B, functional verification

## Abstract

Ethylene response factors (ERFs) are transcription factors that are essential in modulating drought stress responses in plants such as *Arabidopsis* and rice. However, the functional role of ERF in wheat drought stress response remains unclear. We identified 33 wheat *ERF* genes under drought stress using transcriptomic analysis and categorized them into eight subfamilies (I–VIII). Among them, 12 drought-responsive candidate genes were upregulated, and *TaERF13-2B* was selected for further analysis. *TaERF13-2B* overexpression in *Arabidopsis* resulted in significantly reduced survival rates under drought conditions with decreased expression of stress-responsive and antioxidant enzyme genes, indicating that the *TaERF13-2B* gene elevated drought sensitivity in transgenic *Arabidopsis*. In wheat, overexpression of *TaERF13-2B* under drought stress increased malondialdehyde accumulation, decreased chlorophyll and proline levels, and reduced antioxidant enzyme activity. Furthermore, the expression of stress-responsive and antioxidant-related genes was suppressed, suggesting that TaERF13-2B negatively regulates wheat response to drought stress. The interactions between TaERF13-2B and TaCIPK9 were further confirmed using yeast two-hybrid and bimolecular fluorescence complementation. Overall, these discoveries deepen our insights into the wheat ERF family and contribute to the elucidation of the functional role of TaERF13-2B in wheat.

## 1 Introduction

Wheat is as a vital staple crop (Tatham and Shewry, 2008). Abiotic stress factors such as salinity, drought, and extreme temperatures account for a 71% decrease in global wheat yields (Dhakal, 2021), with drought being the leading cause of yield loss (Mwadzingeni et al., 2018). In addition, factors such as soil erosion, nutrient and water depletion, overexploitation of natural vegetation, and environmental disasters intensify the effect of drought stress on wheat production. Transcription factors (TFs) are essential controllers of target gene transcription that bind to *cis*-regulatory elements in promoter genes and are crucial for plant responses to abiotic stress (Saha et al., 2023 4). Although the activity of individual efficient genes can affect the resistance to specific stresses, their overall contribution to abiotic stress tolerance is often limited. In contrast, TFs orchestrate the expression of a suite of stress-responsive genes, enabling plants to tolerate various environmental challenges (Chaudhry et al., 2021). Structurally, TFs constitute a DNA-binding domain, an oligomerization site, a gene regulatory region (which may include activation or repression domains), and a nuclear localization signal. In plants, protein families such as WRKY, bZIP, and ERF are particularly important in regulating stress responses (Gahlaut et al., 2016).

ERFs are a group of TFs exclusive to plants, classified within the APETALA2/ERF (AP2/ERF) family (Li et al., 2024). The ERF subfamily can be categorized further into the ERF and DREB subfamilies (Li et al., 2024). The ERF and DREB subfamilies harbor a single AP2 domain, which features a basic hydrophilic region at the N-terminus comprising three *β*-sheets. The amino acids at positions 14 and 19 in the second *β*-sheet are characteristic of each subgroup: alanine (A) and aspartic acid (D) define the ERF group, whereas valine (V) and glutamic acid (E) define the DREB group (Jiang et al., 2024). ERF-type members bind to GCC-box elements, whereas DREB-type members bind to DRE/CRT elements, thereby regulating the effector genes through activation or repression. ERF serves an essential function in plant growth and development, particularly in regulating plant responses to abiotic stresses, such as drought, salinity, and low temperatures, as well as biotic stresses, including pathogen infections (Debbarma et al., 2019). The effect of ERF on drought tolerance has recently become the focus of research. For example, in maize, overexpression of ERF subfamily members, such as *ZmbZIP72* (Ying et al., 2012), *AtERF74* (Yao et al., 2017; Ma et al., 2024), *ZmEREBP60* (Zhu et al., 2022), and *ZmERF21* (Wang Z. et al., 2022), can enhance drought tolerance. Drought resistance is positively regulated by the OsERF48 (Jung et al., 2017), OsERF71 (Lee et al., 2016), OsERF101 (Jin et al., 2018), OsERF83 (Jung et al., 2021), and OsERF115 (Park et al., 2021) genes in rice; TaERF1 (Xu et al., 2007), TaERF3 (Rong et al., 2014), TaERF87 (Du et al., 2023), and TaERFL1a (Li et al., 2025) in wheat; GmERF9 (Zhai et al., 2017), and GmERF113 (Fang et al., 2022) in soybean, and ERF38 (Cheng et al., 2019), and PtaERF194 (Huan et al., 2023) in poplar, and MhERF113 (Tian et al., 2025) in apple. Under drought stress, certain ERF transcription factors in plants exhibit negative regulatory functions. For instance, under drought stress, *SlERF.B1* overexpression in tomato negatively regulates drought tolerance in transgenic plants by modulating the expression of stress-related genes (Wang Y. et al., 2022). In rice, overexpression of *OsERF109* reduced drought resistance and silencing of the gene enhanced drought resistance (Yu et al., 2017). In *Tamarix hispida*, ThERF1 negatively modulates drought stress tolerance when overexpressed in *Arabidopsis* (Wang et al., 2014). Moreover, AtERF7 negatively regulates drought tolerance by interacting with AtSin3, potentially inducing chromatin modifications and repressing activator binding to DNA through the recruitment of HDA19 (Chen and Wu, 2010). Thus, *ERF* genes are crucial in modulating the responses of plants to drought stressors.

Drought is a key global challenge for agriculture, severely impacting both crop productivity and quality. Wheat has developed complex and intricate regulatory networks for adapting to drought conditions. Although there have been studies on the involvement of plant ERF in controlling drought responses, the drought regulatory network of ERFs in wheat remains unclear. Therefore, further exploration of the genes related to drought stress responses is necessary.

Common wheat is an allopolyploid with an extensive and intricate genome, roughly 1.7 billion base pairs in size, and it contains many genes, which complicates functional studies of its genes. The AP2/ERF family ranks among the largest TF families in plants and it contains a substantial number of the ERFs, making it challenging to rapidly identify the ERF subfamily members associated with drought stress responses in wheat. Therefore, this research focused on utilizing transcriptome sequencing technology to screen for drought-responsive *ERF* genes among the abundant wheat ERF family members, and to characterize the functions of key candidate genes. The findings offer new perspectives on the study of wheat ERF members and provide a theoretical foundation for gene editing in the development of drought-resistant varieties.

## 2 Materials and methods

### 2.1 Plant materials, vectors, and bacterial strains

The spring wheat variety Longmai 26 (LM26) was used to establish a drought-stress transcriptome and amplify gene sequences (*TaERF13-2B*). The spring wheat variety LM26, which is characterized by high yield and high environmental adaptability (Zhang et al., 2006), is widely used in wheat breeding programs. Wheat varieties ‘Fielder’, ‘CB037’, and ‘Westonia’ have been applied extensively in wheat genetic transformation, with ‘Fielder’ exhibiting the highest transformation efficiency (Liu et al., 2023). However, most elite wheat varieties are resistant to genetic transformation and typically exhibit lower transformation efficiency (Luo et al., 2021). Therefore, the present study used the spring wheat cultivar ‘Fielder’ (wild-type, WT) to generate transgenic wheat plants. Due to their distinct varietal characteristics, the spring wheat varieties LM26 and Fielder serve as suitable models for studying the gene of interest. The *Arabidopsis thaliana* ‘Columbia-0’ is a model plant for genetic research and is widely used for gene function validation. In the present study, the WT *Arabidopsis* was used as the receptor material for *TaERF13-2B*.

The vectors used in the present study include the pBI121 *Arabidopsis* overexpression vector (transformation strain GV3101), pGBKT7/PGADT7 vector (transformation strain AH109), pCAMBIA1302-nYFP/cYFP bimolecular-fluorescence complementation (BiFC) vector (transformation strain GV3101), and the pCAMBIA3301 wheat overexpression vector (transformation strain EHA105).

### 2.2 Transcriptomic profiling and phylogenetic assessment of ERF members in wheat

Wheat variety LM26 was cultivated at the two-leaf stage and exposed to drought stress (20% PEG6000). Leaf tissue samples were gathered at 0, 1, 6, and 12 h (Ge et al., 2022; Ye et al., 2021), resulting in 12 wheat samples sent to the Tianjin BMK Company for transcriptome sequencing. The clean reads were quickly and precisely mapped to the reference genome (Version: IWGSC RefSeq v1.1) using HISAT2 software to acquire the localization information of the reads on the reference genome. Subsequently, StringTie was used to assemble the aligned reads and reconstruct the transcriptome for further analysis (SRA: PRJNA1178630). StringTie was used to assemble the transcriptome using a maximum flow algorithm, and fragments per kilobase of transcript per million fragments mapped (FPKM) normalization was applied as an indicator of transcript levels. Differential analysis was conducted using the edgeR software. In the process of identifying differentially expressed genes (DEGs), a fold change ≥ 4 and a false discovery rate < 0.01 were set as the screening criteria. The AP2/ERF family HMM profile (PF00847) from the PFAM was utilized as a query to hunt for and analyze proteins encoded by DEGs using HMMER software (Zhang et al., 2024a). The identified members were assessed through NCBI’s CDD to validate the existence and characterization of the AP2 domain, and candidate ERF members were identified according to the characteristics of the ERF members. Finally, the molecular properties and subcellular localization of each ERF protein were evaluated using the ExPASy and Cell-PLoc software.

All wheat ERF sequence information was obtained from the transcriptome database, whereas the sequence data for *Arabidopsis* and *Brachypodium* were retrieved from Ensembl Plants. The ERF amino acid sequences of these three species were aligned using ClustalX for sequence alignment analysis, and a neighbor-joining (NJ) phylogenetic tree was generated using MEGA7 with 1000 bootstrap replicates.

### 2.3 Characterization of wheat ERF members

Chromosomal location information from the GFF3 file was visualized using TBtools-II (v.2.121). Coding and genomic sequences were submitted to the GSDS database for gene structure analysis, and the gene structure was visualized using the EvolView tool. The XML file for wheat ERF proteins obtained from the MEME database was visualized using TBtools-II (v.2.121). A 1500-bp upstream segment of the ATG from the *TaERF* members was analyzed using PlantCARE to identify *cis*-regulatory elements.

### 2.4 Expression profiling of wheat *ERF* genes

The FPKM values for the wheat *ERF* genes, extracted from the transcriptome data, were visualized using TBtools-II (version 2.121), resulting in the generation of a heat map. The reliability of the transcriptome information was confirmed using qRT-PCR according to methods detailed in our previous studies (Yu et al., 2023). The relative expression levels of the genes were assessed using the 2^−ΔΔCT^ method, with *Actin*-7 (LOC542814) serving as the internal reference gene for wheat. Each experiment was conducted in triplicate. **Supplementary Table S1** lists the primers that were used in this study.

### 2.5 Production of *TaERF13-2B* transgenic plants and the measurement of physiological parameters

The production of *TaERF13-2B* transgenic plants, as described in our previous studies (Yu et al., 2023), resulted in the acquisition of two homozygous T_3_ transgenic *Arabidopsis* lines. Three-week-old *Arabidopsis* seedlings were subjected to a 13-day drought period without water, followed by re-watering for 4 days to analyze drought survival rates. The survival rate was assessed by calculating the ratio of surviving plants to the total number of plants following drought treatment. A plant is considered alive if it has green leaves and exhibits normal growth, while a plant is classified as dead if its leaves are wilted, shriveled, or completely necrotic. One replicate contained 40 seedlings of each line. Three independent survival assays were performed. After a 9-day drought period, physical indices were measured, and the expression levels of stress-related genes were assessed in both WT and overexpressing (OE) plants. Malondialdehyde (MDA), proline (Solarbio, Beijing), and chlorophyll contents were quantified as outlined in previous studies (Yu et al., 2023; El-Esawi et al., 2019), with three biological replicates. Finally, qRT-PCR was performed to measure the mRNA levels of stress-modulated genes (*AtP5CS1*, *AtDREB2A*, *AtRD29A*, *AtCOR15A*, *AtMYB15*, *AtERD10*, *AtLTI30*, *AtKIN1*, and *AtGSTU19*), with three biological replicates analyzed in the transcript profiles of genes.

To construct wheat overexpression (OE) lines, the CDS of *TaERF13-2B* was inserted into the pCAMBIA3301 vector, driven by the maize ubiquitin promoter. *Agrobacterium tumefaciens*-mediated transformation was employed to generate independent transgenic lines for the *TaERF13-2B* construct, using the wheat cultivar Fielder as the genetic background (Ishida et al., 2015). The T_0_, T_1_, and T_2_ transgene-positive plants were detected using PCR, and the T_3_ generation was detected using PCR and qRT-PCR. Wheat seedlings were grown in a controlled environment with a 16 h light/8 h dark cycle and 22°C during the day and 19°C at night. At the two-leaf stage, drought stress was applied by withholding water for 13 days, during which physiological parameters were measured. Drought stress was extended further to 15 days to photograph the drought phenotypes. The measured physiological parameters included chlorophyll content, MDA levels, O_2_^.-^content, and the of antioxidant enzyme activities (SOD, POD, and CAT), according to the methods reported in our previous studies (Yu et al., 2023). Three biological replicates were performed. Quantitative analysis of stress-related genes, including *TaP5CS1*, *TaERF3*, *TaDREB1*, *TaSOD* (*Fe*), *TaPOD*, and *TaCAT*, was performed on day 8 of drought stress, with three biological replicates analyzed in the transcript profiles of genes.

### 2.6 Transcriptional activation analysis and yeast two-hybrid assay

To evaluate the transcriptional activity of TaERF13-2B, the CDS of 290 amino acids, along with truncated fragments representing the N-terminal (1 to 139 aa) and C-terminal (140 to 290 aa) regions, were cloned into the pGBKT7 and introduced into AH109 yeast cells. The transformants were then cultured in the dark at 30°C for 3 days on SD medium deficient in tryptophan and SD medium lacking tryptophan, histidine, and adenine plates to assess their transcriptional activity.

A truncated coding region of TaERF13-2B containing the AP2 domain (amino acids 140–290) was ligated into the pGBKT7 and introduced into yeast cells (AH109) for Y2H screening. Following previously established methods (Yu et al., 2023), a Y2H screening was performed using a cDNA library derived from drought-treated wheat. The positive clones were sequenced for further confirmation. Genes screened from the cDNA library contained full-length CDS and were merged with the pGADT7-prey vector. Modified plasmids carrying the bait TaERF13-2B (140–290 aa) and the pGADT7-prey were co-transformed into AH109 yeast cells. The transformants were then nurtured on solid media double-dropout medium (SD lacking tryptophan and leucine) and quadruple-dropout medium (SD lacking tryptophan, leucine, histidine, and adenine) for point-to-point validation of the Y2H interactions.

### 2.7 Bimolecular fluorescence complementation assay

The complete sequences of TaERF13-2B and TaCIPK9 were individually inserted into pCAMBIA1302-nYFP and pCAMBIA1302-cYFP, respectively. The recombinant vectors were introduced into *A. tumefaciens* and transiently co-expressed in *Nicotiana benthamiana* leaves. Fluorescence signals were visualized using an Olympus IX83 confocal microscope (Olympus, Tokyo, Japan). The specific experimental details can be viewed in our previous study (Yu et al., 2022).

### 2.8 Statistical analysis

Each experiment was conducted with three biological replicates. All data are expressed as mean ± standard error and were statistically analyzed using SPSS 22.0 (IBM SPSS, USA). Group differences were assessed via the Student’s *t*-test, with statistical significance set at *P < 0.05 and highly significant differences at **P < 0.01.

## 3 Results

### 3.1 Identification of wheat ERF members through transcriptomic analysis and their subsequent phylogenetic examination

In a prior study, a transcriptome related to drought stress was established using young leaves from the wheat variety LM26. Transcriptome sequencing was performed on 12 LM26 samples at 0, 1, 6, and 12 h after drought stress, which yielded a large set of DEGs. A local protein database was constructed using the proteins encoded by the DEGs. The HMMER program was used to screen for ERF members, followed by the verification of the presence and integrity of the AP2 domain in the candidate proteins using the NCBI-CDD. This process identified 33 ERFs in wheat (**Supplementary Table S2**). Based on chromosomal distribution analysis, these *TaERF* genes were located on 15 of the 21 wheat chromosomes. The largest number of TaERFs (four) were observed on chromosome 1A. Twelve *TaERF* genes were evenly distributed across chromosomes 1D, 2A, 2B, and 3D, whereas 14 *TaERF* genes were distributed evenly across chromosomes 1 B, 2D, 3A, 3B, 4A, 4B, and 6D. The remaining three *TaERF* genes were individually scattered across the other chromosomes (**Supplementary Figure S1**). Based on their phylogenetic relationships and chromosomal locations, the 33 TaERFs were renamed sequentially from TaERF1-1A to TaERF14-6D. Subsequently, the physical properties of the proteins, including the sequence length, MW, pI, and subcellular localization, were predicted (**Supplementary Table S2**). TaERF8-4D had the longest sequence, consisting of 342 amino acids, whereas TaERF6-3A and TaERF6-3D had the shortest sequences, with 173 amino acids each. TaERF8-4D had the highest MW (36.51 kDa), whereas TaERF6-3A had the lowest (18.12 kDa). The pI values ranged from 4.6 (TaERF8-4D) to 10.07 (TaERF6-3D). Subcellular localization predictions indicated that 33 TaERF proteins were localized in the nucleus.

**Figure 1:**
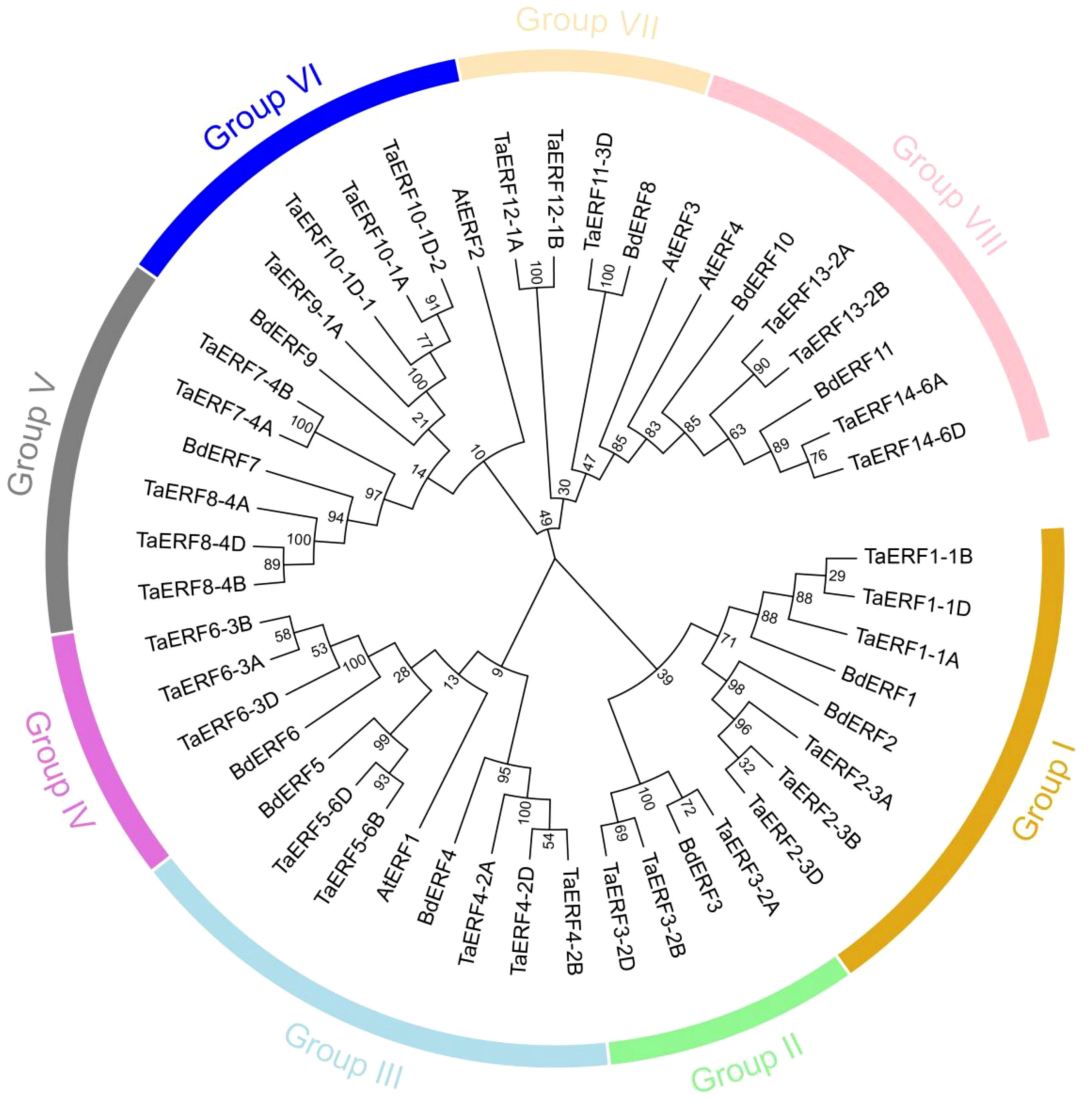
Phylogenetic relationships among ERF members in *Brachypodium*, *Arabidopsis*, and wheat. The wheat ERF proteins were grouped into eight major clades (Groups I–VIII), each highlighted with a distinct color for clear visualization.

An NJ phylogenetic tree was constructed using 33 wheat, 11 *Brachypodium*, and four *Arabidopsis* ERFs to explore the phylogenetic ties within the TaERF members (**Figure 1**; **Supplementary Table S3**). The 33 wheat TaERFs were classified into eight phylogenetic groups: Groups I–VIII, containing six, three, five, three, five, four, three, and four TaERF proteins, respectively.

### 3.2 Gene organization, conserved motifs, and domain characterization of wheat ERFs

**Figure 2:**
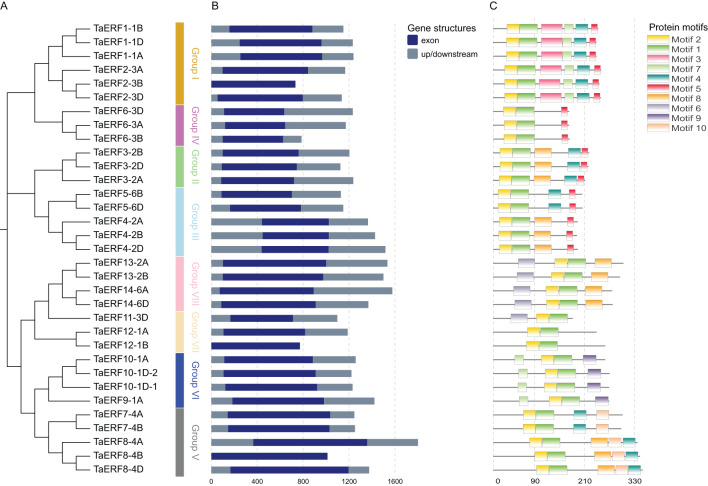
NJ phylogenetic tree, intron-exon organization, and conserved motifs of wheat ERFs. **(A)** NJ phylogenetic tree of TaERFs. **(B)** Structures of *ERF* genes in wheat. **(C)** Arrangement of conserved motifs within ERF proteins.

Based on the NJ phylogenetic tree, ERFs in wheat were classified into eight groups: I–VIII (**Figure 2A**). The intron-exon structure provides insights into the structural features of the ERF family (**Figure 2B**). All ERF members lacked introns, indicating the evolutionary conservation of this family. Transcriptional output may be delayed by the presence of numerous and lengthy introns, potentially leading to the suppression of gene expression under adverse conditions. In contrast, genes with fewer or shorter introns are likely to exhibit more efficient expression when responding to stress environments (Heyn et al., 2015). Thus, the absence of introns in ERFs may facilitate rapid responses to abiotic stress conditions in wheat.

Potential motifs in all TaWRKY proteins were predicted using MEME. Ten conserved motifs (motifs 1–10), ranging in size from 15 to 49 aa, were identified (**Supplementary Figure S2**). The ERFs belonging to a similar subgroup exhibited similar motif compositions. For example, the six protein sequences of Group I included motifs 1, 2, 3, 4, 5, and 7 (**Figure 2C**). Motifs 1 and 2 were present in all ERFs and encoded the AP2 domain (**Supplementary Figure S3**). In the AP2 domain, the 14th residue was alanine, and the 19th was aspartic acid (**Supplementary Figure S3**), confirming that all 33 genes are typical ERFs.

### 3.3 *cis*-acting element analysis of ERF members in wheat

**Figure 3:**
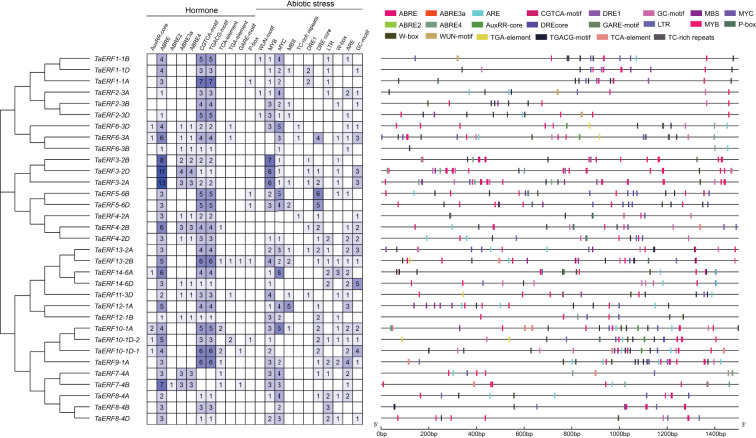
Predicted *cis*-regulatory elements in the 1500-bp promoter fragments of wheat ERF members.

Using PlantCARE, *cis*-regulatory elements in *TaERF* promoters were predicted, revealing numerous elements linked to hormone signaling and responses to abiotic stress (**Figure 3**). The identified hormone-related elements encompassed a variety of responsive motifs, including auxin-regulated elements (TGA element and AuxRR core), abscisic acid-activated elements (ABRE), methyl jasmonate-regulated elements (CGTCA and TGACG), salicylic acid-associated elements (TCA element), and gibberellin-associated elements (GARE-motif and P-box). In addition, several abiotic stress-associated elements were observed, including the mechanical injury response element (WUN-motif), binding sites for MYB, MYC, and WRKY TFs (MYB, MYC, and W-box), defense and stress-associated elements (TC-rich repeats), drought-inducible element (MBS), dehydration-responsive elements (DRE), anaerobic induction elements (ARE), low-temperature-regulated elements (LTR), and hypoxia-specific-associated elements (GC-motif). The results imply that *TaERF* genes are essential for the regulation of wheat growth and development, as well as its adaptation to environmental factors.

### 3.4 Analysis of wheat *ERF* genes under drought conditions

**Figure 4:**
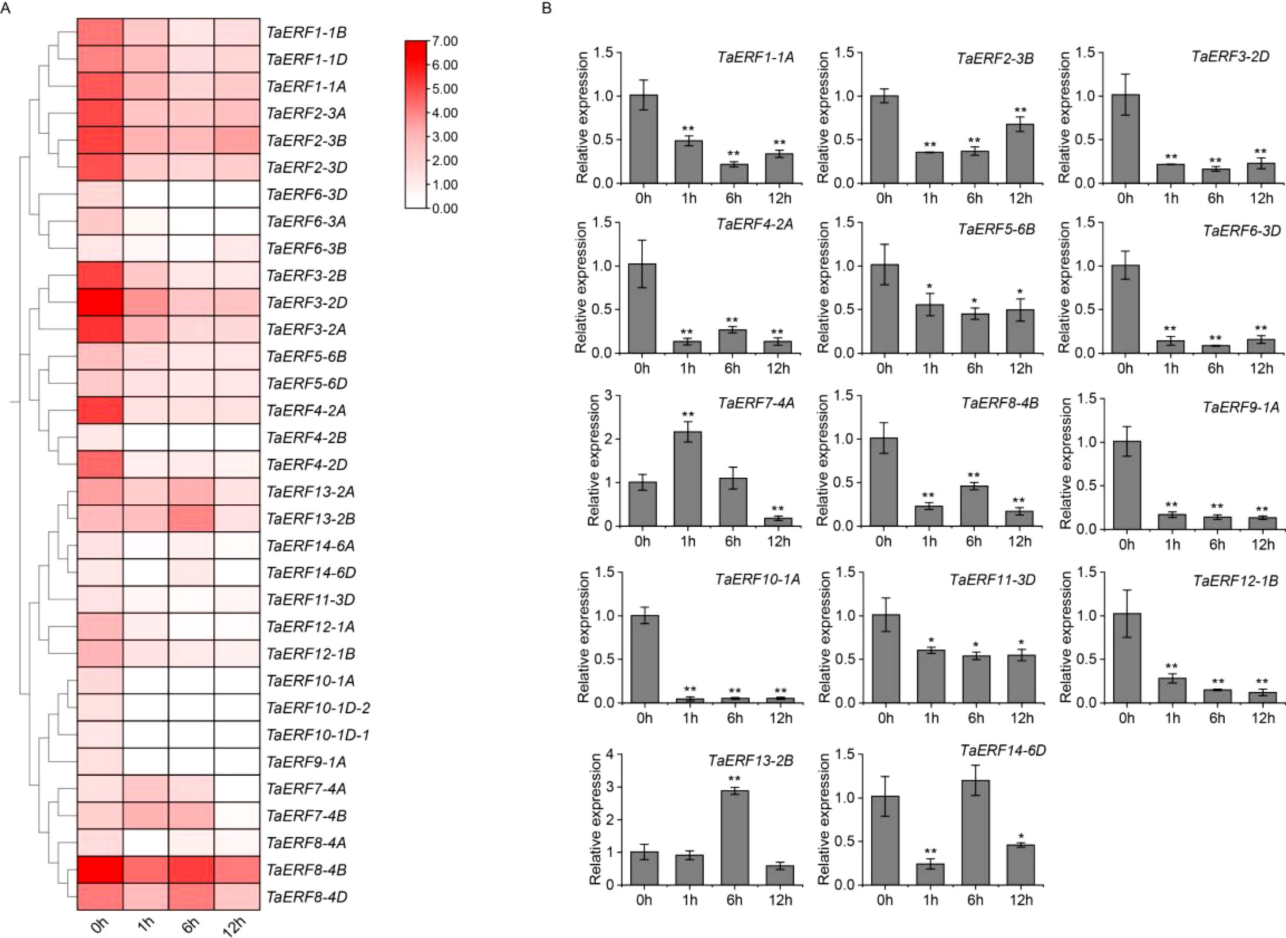
RNA-seq profiling and qRT-PCR confirmation under drought stress. **(A)** Heatmap illustrating the transcript abundance of 33 wheat *ERF* genes in LM26 leaves before and after drought stress. The spring wheat variety LM26 was used to establish a drought-stress transcriptome. The gradient color scale ranges from white (low expression) to red (high expression), visually representing the variations in expression levels. The data are derived from RNA-seq analysis, represented as FPKM values, which mirror the relative transcript expression levels. **(B)** Expression levels of 14 TaERF members in wheat variety LM26 under drought treatment assessed using qRT-PCR. Experiments were analyzed for three biological replicates. Statistical significance was assessed using Student’s *t*-test, with *P* < 0.05 indicated by (*) and *P* < 0.01 by (**).

To gain insights into the biological roles of wheat ERF members, we assessed the fragments per kilobase of transcript per million fragments mapped (FPKM) values of 33 *ERF* DEGs identified from RNA-seq data collected before and following drought stress, and visualized the results (**Figure 4A**). The common wheat (*Triticum aestivum* L.) is an allohexaploid plant. Most wheat genes exist in three copies and exhibit a high degree of sequence identity within their coding regions, suggesting potential functional similarity. In the present study, under drought stress, most homologous genes displayed similar expression patterns (**Figure 4A**). For example, the *TaERF3* gene (*TaERF3-2A*, *TaERF3-2B*, and *TaERF3-2D*) was significantly downregulated under drought stress. Similarly, the *TaERF14* gene (*TaERF14-6A* and *TaERF14-6D*) exhibited an initial downregulation at 1 h, followed by upregulation at 6 h, and a subsequent downregulation at 12 h. Thus, one homolog from the TaERF1~14 gene group was selected randomly for qRT-PCR validation. As illustrated in **Figure 4B**, the expression patterns obtained from qRT-PCR closely matched the transcriptome information, confirming the accuracy of the RNA-seq results. Most wheat *ERF* genes were downregulated markedly under drought conditions. Examples include *TaERF1-1A*, *TaERF2-3B*, *TaERF3-2D*, *TaERF4-2A*, *TaERF5-6B*, *TaERF6-3D*, *TaERF8-4B*, *TaERF9-1A*, *TaERF10-1A*, *TaERF11-3D*, and *TaERF12-1B*. In contrast, *TaERF7-4A* was significantly upregulated after 1 h of exposure to drought stress, recovered to baseline levels after 6 h, and was further downregulated at 12 h. *TaERF13-2B* was significantly upregulated at 6 h, followed by a return to baseline levels at 12 h. Meanwhile, *TaERF14-6D* was significantly downregulated after 1 h, recovered to basal levels, and was subsequently downregulated.

### 3.5 Functional analysis of *TaERF13-2B* in *Arabidopsis* under drought stress

**Figure 5:**
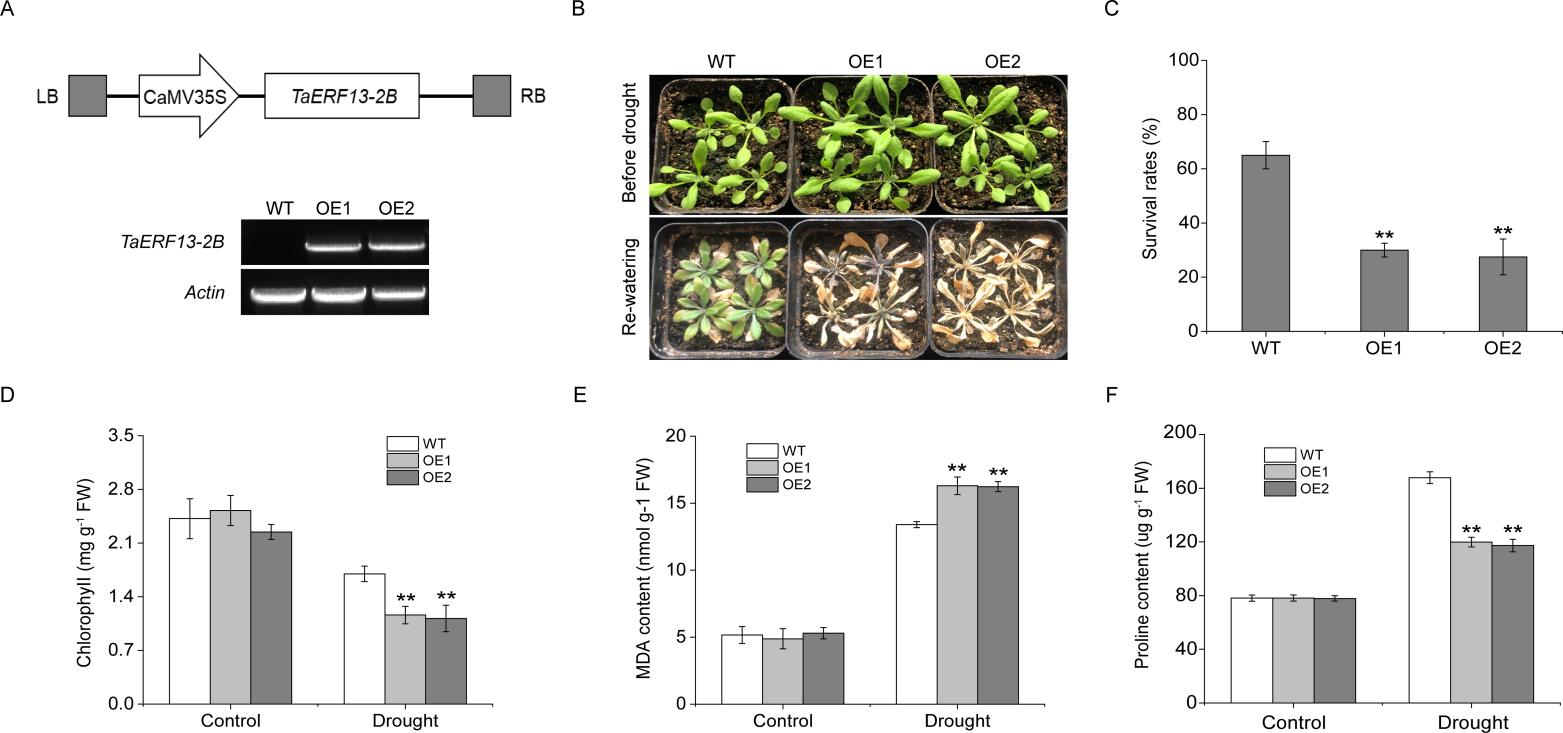
Functional analysis of *TaERF13-2B* overexpression lines in *Arabidopsis* under drought conditions. **(A)** Schematic representation of the *TaERF13-2B* plant overexpression vector and RT-PCR detection. **(B)** Photographs of *Arabidopsis* plants taken before drought stress and after 13 days of drought followed by 4 days of re-watering, along with the calculation of survival rates **(C)**. Measurements of physiological parameters under drought stress, including chlorophyll content **(D)**, MDA levels **(E)**, and proline content **(F)**. The T_3_ generation transgenic *Arabidopsis* lines used in the experiment were OE1 and OE2. The experiments were analyzed for three biological replicates. Statistical significance was assessed using Student’s *t*-test, with *P* < 0.01 indicated by (**). Scale bars: 3.5 cm.

Among the 33 *ERF* DEGs, *TaERF13-2B* showed the largest drought-induced transcript-level differences, with a 2.83-fold upregulation after 6 hours of drought stress, and its promoter region contained abundant stress-related *cis*-acting elements, suggesting that TaERF13-2B may serve an essential function in regulating drought stress responses. Therefore, the gene was selected for further investigation. To determine the function of TaERF13-2B, an OE vector for this gene was constructed in *Arabidopsis*. Two homozygous T_3_ transgenic plants were obtained, and their *TaERF13-2B* expression levels were analyzed using RT-PCR (**Figure 5A**). The findings revealed that *TaERF13-2B* was not expressed in the WT plants, whereas it was detected successfully in the OE plants, confirming the effective introduction of *TaERF13-2B* into *Arabidopsis*. Before the drought stress, no notable differences were recognized in growth between the WT and OE plants (**Figure 5B**). After 13 days of water deprivation followed by 4 days of re-watering, most WT plants resumed growth, albeit slower, whereas most OE lines died, with leaves exhibiting chlorosis. Survival rate analysis further confirmed that the survival rate of OE plants was markedly lower than that of the WT plants (**Figure 5C**). Under normal watering treatments, no significant differences were recognized in chlorophyll content (**Figure 5D**), MDA levels (**Figure 5E**), or proline content (**Figure 5F**) between the WT and OE plants. However, under drought conditions, chlorophyll and proline contents were markedly lower in the OE plants, whereas MDA levels were markedly higher than those in the WT plants.

**Figure 6:**
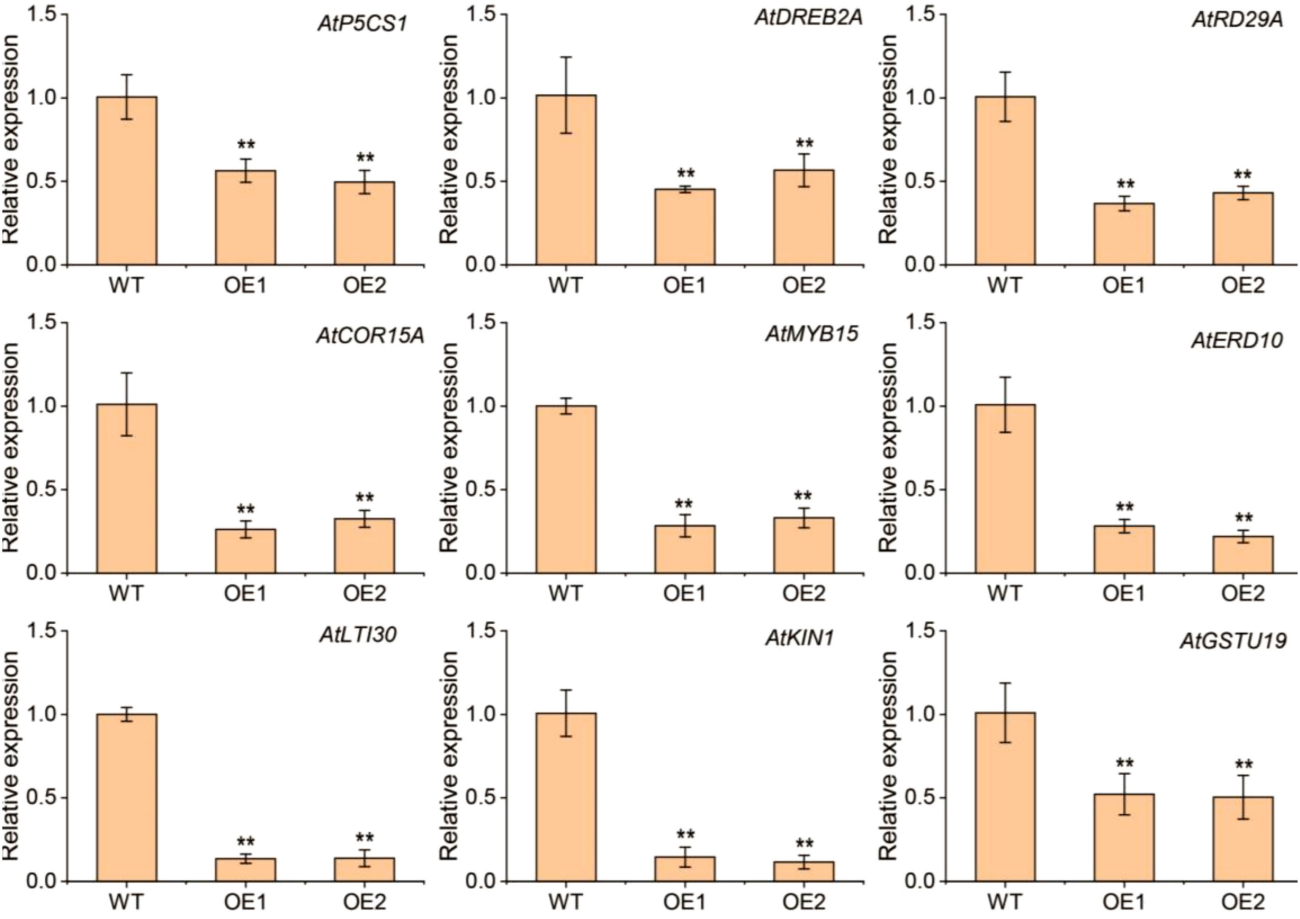
Transcription levels of nine genes associated with stress responses—*AtP5CS1*, *AtRD29A*, *AtDREB2A*, *AtCOR15A*, *AtMYB15*, *AtERD10*, *AtLTI30*, *AtKIN1*, and *AtGSTU19*—were measured in *TaERF13-2B Arabidopsis* overexpression (OE) lines (T_3_ generation transgenic lines: OE1 and OE2) under drought stress. Three biological replicates were conducted. Student’s *t*-test was performed (^**^ *P* < 0.01).

Under drought conditions, the transcript levels of nine stress-responsive genes—*AtP5CS1*, *AtRD29A*, *AtDREB2A*, *AtCOR15A*, *AtMYB15*, *AtERD10*, *AtLTI30*, *AtKIN1*, and *AtGSTU19*—were assessed in the OE plants using qRT-PCR (**Figure 6**). In comparison to the WT plants, the transcript levels of these genes were lower in the *TaERF13-2B* OE plants. The findings reveal that *TaERF13-2B* OE in *Arabidopsis* may alter physiological traits by modulating the expression of stress-associated genes, thereby increasing the sensitivity of transgenic plants to drought conditions.

### 3.6 Functional analysis of *TaERF13-2B* in wheat under drought conditions

**Figure 7:**
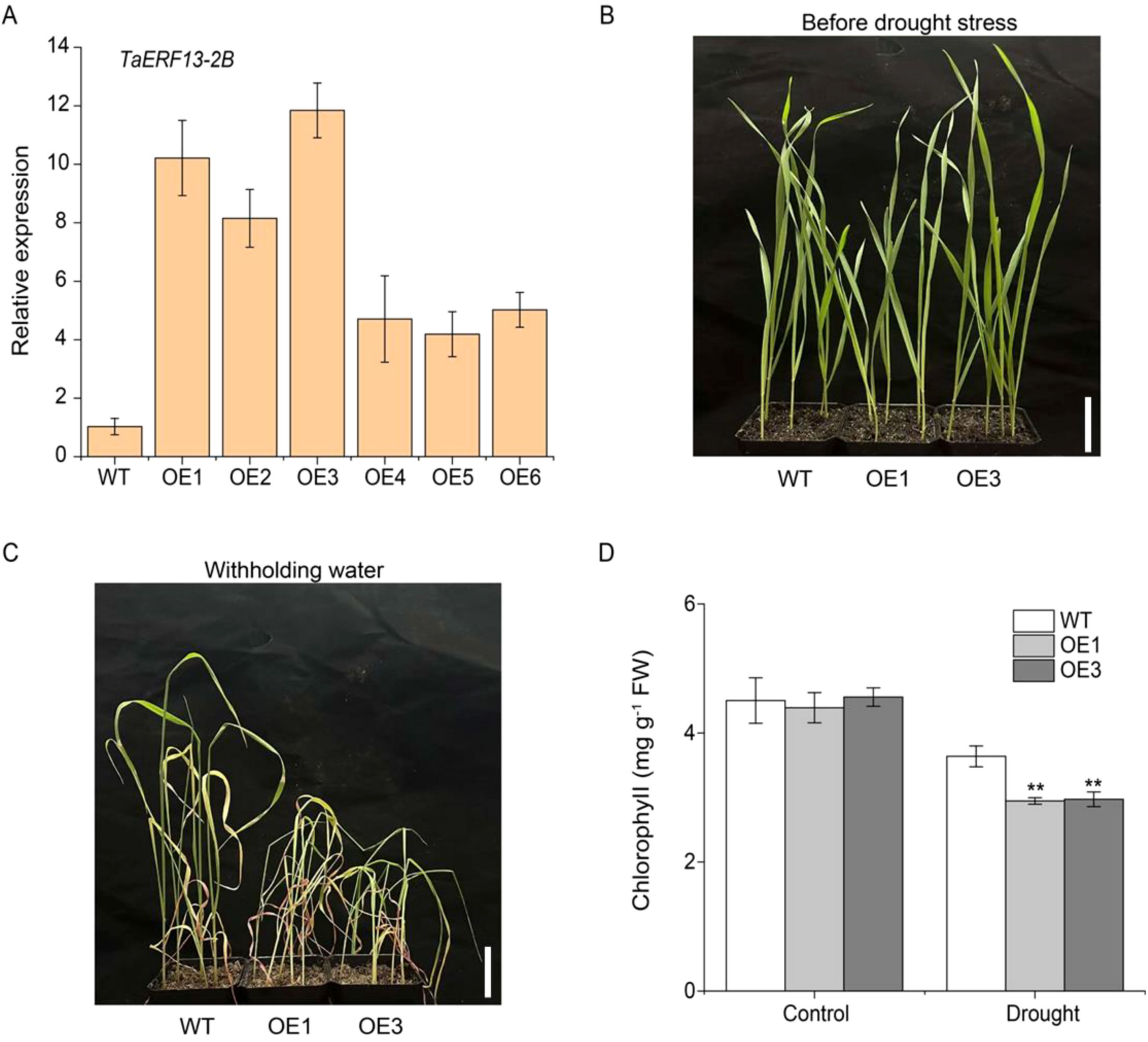
Functional analysis of *TaERF13-2B* transgenic wheat under drought stress. **(A)** Transcription level analysis of *TaERF13-2B* in transgenic wheat lines. **(B)** Phenotypic analysis of transgenic wheat seedlings before drought stress. **(C)** Phenotypic analysis of transgenic wheat seedlings under drought stress. **(D)** Chlorophyll content in wheat leaves after drought stress. Three biological replicates were conducted. Asterisks indicate significant differences relative to the WT line (Student’s *t*-test: **P < 0.01). Scale bars: 3.5 cm.

**Figure 8:**
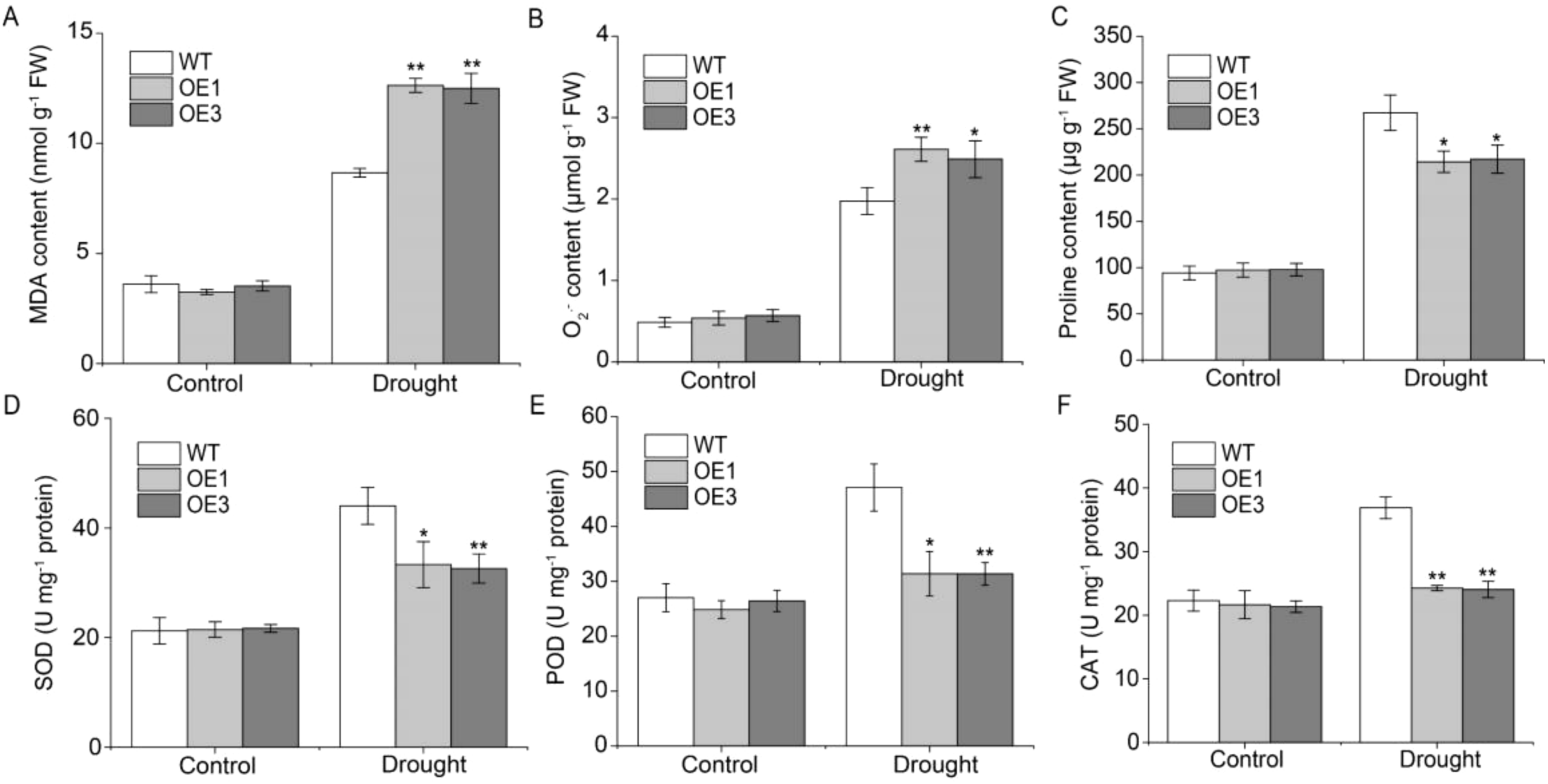
Measurements of physiological parameters in transgenic wheat under drought conditions, including the concentrations of MDA **(A)**, O_2_^.-^ **(B)**, and proline **(C)**, and the activities of SOD **(D)**, POD **(E)**, and CAT **(F)**. Three biological replicates were conducted. Asterisks indicate significant differences relative to the WT line (Student’s *t*-test: *P < 0.05; **P < 0.01).

To examine TaERF13-2B function in wheat under drought conditions, the *TaERF13-2B* gene were overexpressed in wheat and further validated its regulatory role in the drought stress response using stable *TaERF13-2B* OE lines. The expression levels of *TaERF13-2B* in the OE wheat plants were measured using qRT-PCR (**Figure 7A**). Compared with the control, the expression levels of *TaERF13-2B* were upregulated markedly in all six OE plants, with the OE3 line exhibiting the most pronounced increase. We selected the homozygous transgenic lines with higher expression levels of OE1 and OE3 for further studies. Before drought stress, leaf morphology between WT and transgenic lines at the seedling stage was not different (**Figure 7B**). After 15 days under drought conditions, the OE wheat plants wilted severely, whereas drought stress had a comparatively mild impact on the WT (**Figure 7C**). The effect of drought stress on the physiological traits of the transgenic wheat indicated that under drought stress, the chlorophyll (**Figure 7D**) and proline (**Figure 8C**) contents, as well as SOD (**Figure 8D**), POD (**Figure 8E**), and CAT activities (**Figure 8F**), were markedly lower in the OE plants than in the WT ones. Conversely, the MDA (**Figure 8A**) and O_2_^.-^ (**Figure 8B**) contents were notably increased notably in the OE plants compared to in the WT plants. In summary *TaERF13-2B* gene overexpression in wheat under drought conditions improved the sensitivity of OE plants markedly.

**Figure 9:**
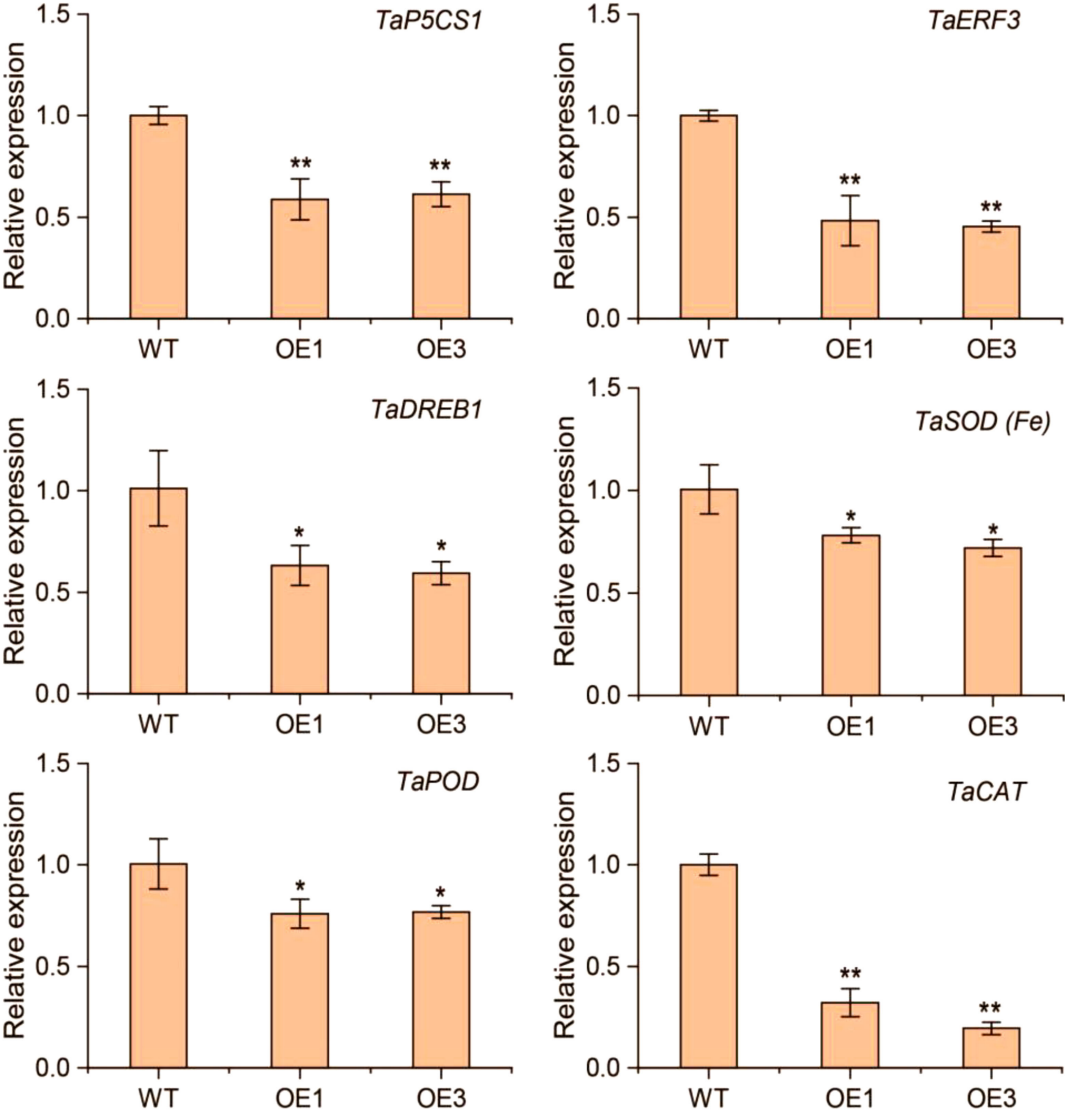
Transcript levels of six stress-associated genes—*TaP5CS1*, *TaERF3*, *TaDREB1*, *TaSOD* (*Fe*), *TaPOD*, and *TaCAT*—were measured in *TaERF13-2B* overexpressing lines under drought stress. Three biological replicates were conducted. Student’s *t*-test was performed (*P < 0.05; **P < 0.01).

We further analyzed the expression levels of *TaP5CS1*, *TaERF3*, *TaDREB1*, *TaSOD* (*Fe*), *TaPOD*, and *TaCAT* in the *TaERF13-2B* wheat OE lines following drought stress (**Figure 9**). Under drought conditions, all stress-related genes were downregulated markedly, with the most pronounced downregulation observed in *TaCAT*. The results imply that TaERF13-2B may regulate the transcript levels of stress-associated genes in wheat during drought conditions, triggering shifts in the physiological traits of seedlings and ultimately increasing susceptibility of transgenic wheat to drought conditions.

### 3.7 Transcriptional activity and protein interaction analysis of TaERF13-2B protein

**Figure 10:**
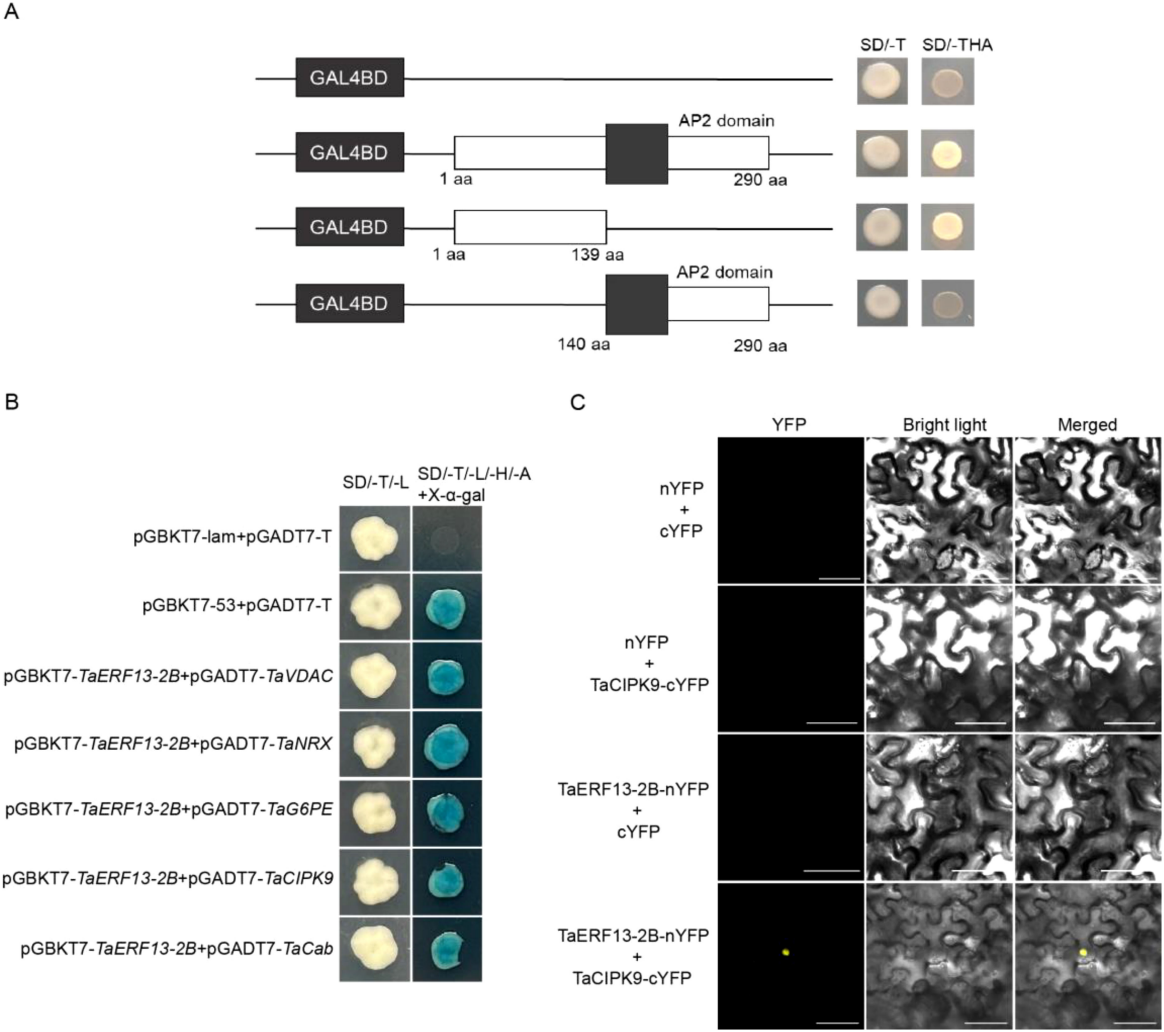
Transactivation activity and Y2H assay analysis. **(A)** Analysis of the transcriptional activity of the TaERF13-2B protein. **(B)** Yeast point-to-point verification of the interaction between TaERF13-2B and candidate proteins. **(C)** Bimolecular-fluorescence complementation (BiFC) assay of TaERF13-2B with TaCIPK9. The complete sequences of *TaERF13-2B* and *TaCIPK9* were inserted into the vectors pCAMBIA1302-nYFP-N1 and pCAMBIA1302-cYFP-N1, respectively. These recombinant plasmids were introduced into tobacco leaves and transiently expressed through agroinfiltration. Scale bars: 50 μm.Y2H: yeast two-hybrid.

To facilitate subsequent Y2H studies, the transcriptional activity of TaERF13-2B was analyzed. Full-length *TaERF13-2B* (1–290 aa) and two truncated bait constructs, *TaERF13-2B* (1–139 aa) and *TaERF13-2B* (140–290 aa), were generated. These recombinant vectors, along with an empty pGBKT7 control vector, were introduced into yeast cells. Yeast strains carrying pGBKT7-*TaERF13-2B* (140–290 aa) grew only on the SD medium lacking tryptophan (SD/-Trp) (**Figure 10A**), indicating the absence of transcriptional activation. In contrast, strains transformed with either pGBKT7-*TaERF13-2B* (1–290 aa) or pGBKT7-*TaERF13-2B* (1–139 aa) showed robust growth in the three-dropout (-Trp/-His/-Ade) medium (**Figure 10A**). These findings suggest that full-length TaERF13-2B exhibits transcriptional activation activity, with the N-terminal (1–139 aa) region identified as the transcriptional activation domain.

A Y2H screen was performed using a wheat cDNA library to identify interacting proteins. A few yeast colonies grew on double-dropout (-Trp/-Leu) and quadruple-dropout (-Trp/-Leu/-His/-Ade) media. Redundant genes were removed, and the remaining candidate interacting proteins were annotated using the NCBI database. Five potentially interacting proteins were identified: TaVDAC, TaNRX, TaG6PE, TaCIPK9, and TaCAB; detailed annotations are provided in **Supplementary Table S4**. The genes encoding these candidate proteins contained complete ORFs and were cloned into the pGADT7 vector to construct prey plasmids for Y2H reverse validation. Each of the five pGADT7 recombinant vectors was co-transformed with pGBKT7-TaERF13-2B (aa 1–334) into the Y2H yeast strains. All transformed strains exhibited growth on both the SD medium lacking tryptophan and leucine (SD/-Trp/-Leu), and the SD medium lacking tryptophan, leucine, histidine, adenine, and supplemented with X-α-Gal (SD/-Trp/-Leu/-His/-Ade/+X-α-Gal) (**Figure 10B**). Overall, the results confirm that TaERF13-2B interacts with five putative proteins in the yeast system. Studies have demonstrated that CIPK9 is crucial in regulating plant adaptations to drought conditions (Song et al., 2018; Shabala et al., 2021). To investigate the interaction between TaERF13-2B and TaCIPK9 in a specific cellular location, both were co-expressed in tobacco plants. YFP fluorescence was detected upon co-expression of *TaERF13-2B* and *TaCIPK9*, while no fluorescence was observed in the control groups **Figure 10C**. This confirmed the interaction between TaERF13-2B and TaCIPK9 in live plant cells through BiFC analysis.

## 4 Discussion

As the largest group within the AP2/ERF family, the ERF subfamily is a critical component regulating how plants respond to abiotic stress. However, owing to the intricacy of the wheat genome, research on the involvement of the ERF response to environmental stress remains limited. In the present study, we identified an *ERF* gene, *TaERF13-2B*, in a transcriptome database. This gene lacks introns, suggesting that *TaERF13-2B* responds rapidly to drought stress. qRT-PCR analysis revealed that the transcription of *TaERF13-2B* in the wheat variety LM26 was significantly induced under drought stress. Furthermore, promoter analysis indicated the occurrence of diverse abiotic stress-responsive elements in the *TaERF13-2B* promoter. The findings indicate that TaERF13-2B is a pivotal regulator of wheat adaptation to drought stress. The AP2 domain of TaERF13-2B is highly conserved, with the 14th and 19th amino acid residues aligned with the characteristics of typical ERFs in plants. Functional analysis demonstrated that the N-terminal region of TaERF13-2B exhibited transcriptional activation activity, whereas the C-terminal region lacked this capability. The results confirmed that *TaERF13-2B* is a typical *ERF* gene capable of regulating transcription in response to drought stress.

Wheat *TaERF13-2B* overexpression in *Arabidopsis* revealed that OE lines exhibited increased sensitivity to drought stress. Earlier research has indicated that the stress-resistance functions of transcription factors may be conserved across heterologous and homologous crops (Li et al., 2017, 2020). For instance, transgenic *Arabidopsis* and rice plants expressing the rice *OsDREB1F* have enhanced tolerance to salt, drought, and low temperatures in *Arabidopsis* and rice (Wang et al., 2008). We validated the drought-related function of *TaERF13-2B* in wheat. *TaERF13-2B* overexpression in wheat caused enhanced drought tolerance markedly during the seedling stage of transgenic wheat plants. The findings reveal that the role of wheat TaERF13-2B in the drought stress response may be conserved across *Arabidopsis* and wheat during the seedling stage, providing an excellent genetic resource for drought-resistant breeding through gene editing in wheat.

Drought stress disrupts the redox balance in plants, resulting in the accumulation of excessive reactive oxygen species (ROS), including superoxide anions, hydrogen peroxide, hydroxyl radicals, and singlet oxygen (Rahman et al., 2024). Plants rely on primarily antioxidant enzyme systems to regulate ROS contents and protect cells from oxidative damage (Ben Saad et al., 2024). ROS can react with polyunsaturated fatty acids (PUFAs), such as linoleic and linolenic acids, in membranes, triggering lipid peroxidation and producing MDA and other aldehydes. MDA can crosslink with membrane proteins and phospholipids, compromising membrane integrity and increasing membrane permeability (Lee and Kim, 2024). Elevated MDA levels indicate significant oxidative damage in plants and are widely used as indicators to assess stress severity (Samanta et al., 2024; Raza et al., 2024). The effectiveness of a plant’s antioxidant protection system can be evaluated by measuring MDA levels and antioxidant enzyme activities (SOD, CAT, and POD) (Zhang et al., 2024b). Additionally, proline is a critical osmoprotectant, helping cells maintain turgor pressure and metabolic activities under stress conditions (Koc et al., 2024). Proline contributes to ROS scavenging and redox homeostasis within cells (Akram et al., 2024). Compared to well-watered conditions, proline accumulation increased in both *Arabidopsis* and wheat under drought stress. However, the magnitude of this increase was lower in transgenic plants than in WT plants, with transgenic lines exhibiting significantly reduced proline levels under stress. In plants, there is often a trade-off between growth and stress tolerance. Overexpression of certain genes, particularly those that promote growth and development, may enhance plant growth while compromising drought resistance. For instance, ectopic expression of *MdZAT10* has been shown to promote seed germination and seedling growth in *Arabidopsis*, but at the cost of reduced drought tolerance (Yang K. et al., 2021). In this study, the lower proline accumulation in transgenic plants under drought stress may be attributed to the specific regulatory function of TaERF13-2B, though further investigation is required to elucidate the underlying mechanisms. Under drought stress, wheat plants overexpressing the *TaERF13-2B* gene exhibited elevated levels of MDA and O_2_, along with reduced proline buildup and POD, CAT, and SOD activities. Additionally, drought stress markedly downregulated the transcript levels of stress-responsive (*TaERF3* and *TaDREB1*), antioxidant-related (*TaSOD (Fe)*, *TaPOD*, and *TaCAT*), and proline biosynthesis genes (*TaP5CS1*) in the transgenic plants. These findings indicate that TaERF13-2B may regulate the expression of multiple gene categories, altering the physiological characteristics of transgenic lines under stress and increasing their sensitivity to drought. Under drought stress conditions, the root system, as the primary organ in direct contact with the soil, undergoes significant morphological changes that influence water use efficiency and subsequently affect crop physiological traits (Hou et al., 2023). In this study, multiple physiological parameters in transgenic plants exhibited notable changes under drought stress compared to the wild type. Based on these findings, we hypothesize that TaERF13-2B may regulate root development and architecture, thereby affecting water use efficiency and playing a role in the drought stress response in wheat.

To further elucidate the mechanism by which TaERF13-2B responds to drought stress, we used a Y2H system to identify the five interacting proteins. The identified proteins were the voltage-dependent anion channel (VDAC), nucleoredoxin (NRX), glucose-6-phosphate 1-epimerase (G6PE), CBL-interacting protein kinase 9 (CIPK9), and chlorophyll a/b binding protein (CAB) (**Supplementary Table S4**). VDAC is essential for the regulation of plant responses to abiotic stress. As a key protein in the mitochondrial outer membrane, it modulates metabolite exchange, ROS homeostasis, and stress signaling, which are vital for plants to tolerate environmental stress (Yang M. et al., 2021; Yu et al., 2022). For example, wheat TaVDAC1 enhances salt tolerance but reduces drought resistance in *Arabidopsis* by regulating ROS homeostasis (Yu et al., 2022). In addition, NRX is a redox-regulating protein belonging to the thioredoxin family. They are essential for cellular redox homeostasis through modulation of the redox state of proteins, which is critical for abiotic stress. NRX contributes to drought tolerance by maintaining membrane integrity and controlling ROS levels (Dos Santos and Rey, 2006; Davoudi et al., 2022; Hazra et al., 2024). G6PE serves a vital function in carbohydrate metabolism, contributing to energy production and normal cellular metabolism (Zhang et al., 2023). Additionally, G6PE may facilitate plant responses to oxidative and environmental stress by regulating their cellular redox states (Zhang et al., 2023). CBL-interacting protein kinases (CIPKs) are critical components of plant signaling networks, particularly in response to salinity and drought stress (Chen et al., 2024). CAB is an essential component of photosynthetic processes in plants, algae, and some bacteria. They serve as key factors in photosynthesis by binding to chlorophyll and facilitating light absorption, transforming solar energy into biochemical energy, which is fundamental to plant health and growth as it enables efficient energy utilization and supports various physiological functions necessary for survival and development. Therefore, TaERF13-2B may interact with five proteins involved in metabolic pathways, signal transduction, redox regulation, and photosynthesis in wheat, thereby controlling the activity of genes involved in downstream processes that influence drought resistance in wheat. In *Arabidopsis*, the *cipk9* mutant exhibited enhanced drought resistance compared to the control under drought stress. Similarly, in rice, the *cipk9* mutant demonstrated greater tolerance under salt and osmotic stress. The findings indicate that *CIPK9* plays a crucial role in response to drought stress. Therefore, we selected the wheat *CIPK9* for further investigation. Using the BiFC assay, we verified the interaction between TaERF13-2B and CIPK9 in plant cells and observed that their interaction occurs in the nucleus. We acknowledge the importance of elucidating the relationship between TaERF13-2B and its downstream targets to better understand the molecular mechanisms underlying drought response. In this study, we performed qRT-PCR analysis of stress-related genes and found that their expression levels were affected in transgenic lines. Further analysis of the promoter elements of these genes is required, followed by promoter binding assays to identify potential downstream targets. Future research will also focus on elucidating CIPK9 function in wheat under drought stress and, how the interaction between TaERF13-2B and CIPK9 regulates the expression of downstream target genes in response to drought stress.

## 5 Conclusion

The present study identified 33 members of the wheat ERF family from the drought stress transcriptome and classified them into eight groups. Analyses of gene expression profiles and *cis*-acting elements indicated that TaERFs are strongly linked to responses to environmental stress. Phenotypic analysis of transgenic *Arabidopsis* and wheat plants overexpressing *TaERF13-2B* under drought conditions revealed that the gene increased sensitivity to drought. Alterations in various physiological parameters and stress-associated gene expression further supported the finding. We demonstrated that TaERF13-2B interacts with multiple proteins and participates in processes related to metabolism, photosynthesis, redox regulation, and signal transduction. This research deepens our knowledge of the wheat ERF TFs and offers important insights for further functional research on *ERF* genes in wheat.

## Data Availability

The original contributions presented in the study are included in the article/**Supplementary Material**. Further inquiries can be directed to the corresponding authors.
