# Supplementary material for: The ERF transcription factor TaERF13-2B functions as a negative regulator of drought tolerance in Arabidopsis and wheat
Source: Front Plant Sci. 2025 Mar 27;16:1535850. doi: 10.3389/fpls.2025.1535850 (PMC11983610; doi:10.3389/fpls.2025.1535850)
Supplement: Supplementary file 1 [file DataSheet1.docx]

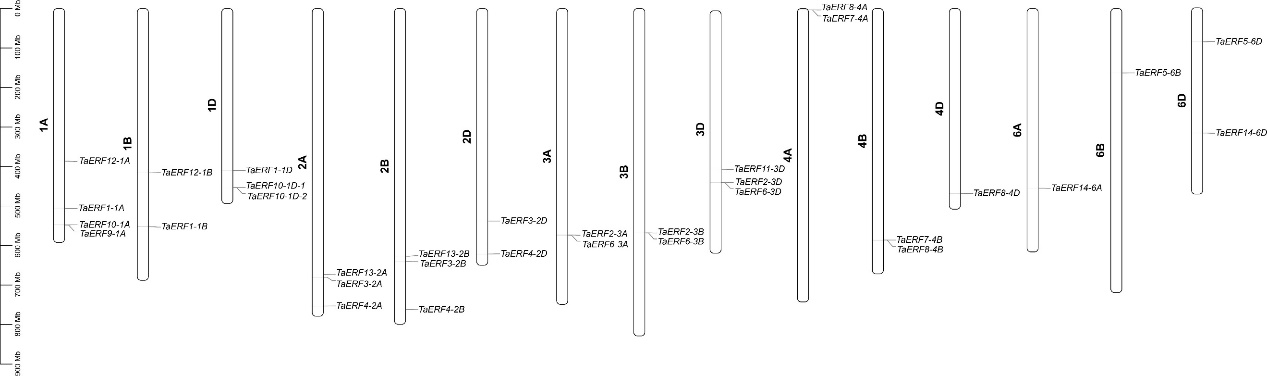


Figure S1. Chromosome distribution of *ERF* genes in wheat.


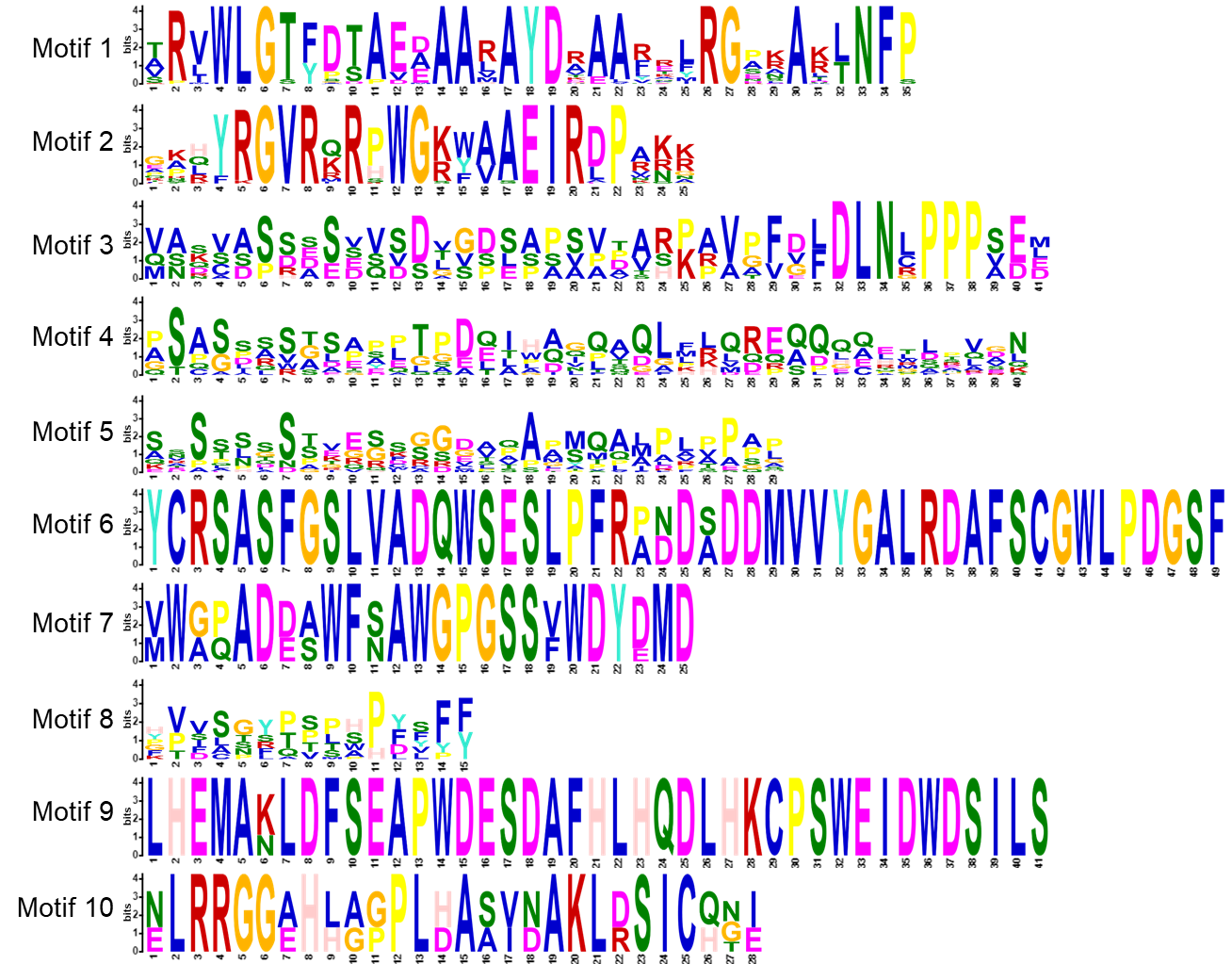


Figure S2. Analysis of conserved motifs in TaERF proteins.


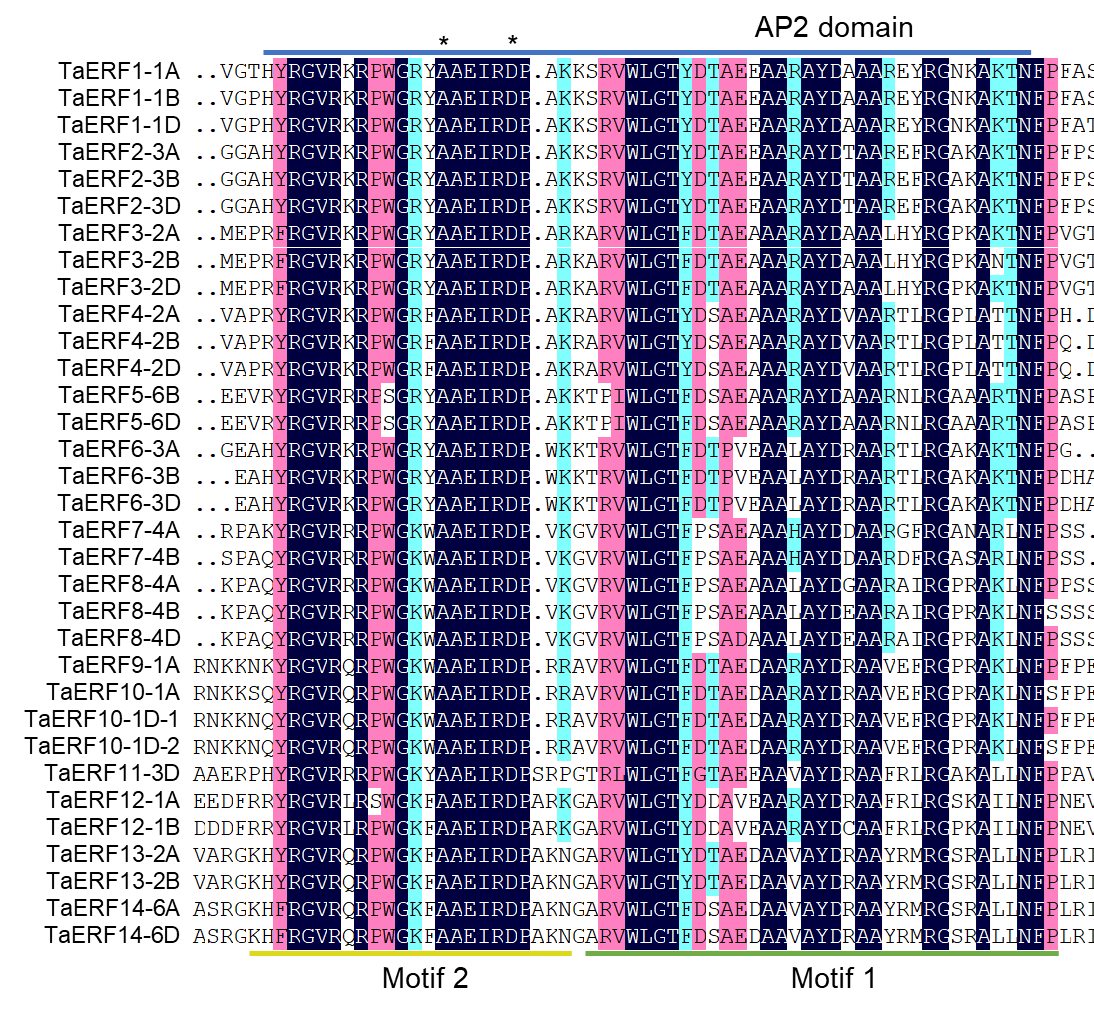


Figure S3. Conserved AP2 domain of TaERF proteins. Asterisks Indicate the Key 14th and 19th residues.
