# Supplementary material for: The ERF transcription factor TaERF13-2B functions as a negative regulator of drought tolerance in Arabidopsis and wheat
Source: Front Plant Sci. 2025 Mar 27;16:1535850. doi: 10.3389/fpls.2025.1535850 (PMC11983610; doi:10.3389/fpls.2025.1535850)
Supplement: Supplementary file 2 [file DataSheet2.docx]

| Table S1. Primer pairs used in the present study | | |
| --- | --- | --- |
| Primer | Usage | Sequence (5'-3') |
| TaERF1-1A F | qRT-PCR | GTACTACTTCTTCGGGCAGGC |
| TaERF1-1A R |  | AGTTCAGGTCGAGGTCGAACG |
| TaERF2-3B F |  | CCAAGGCGAAAACTAACTTCCCG |
| TaERF2-3B R |  | TAGAGTCCAAGGTGCTGTTGCT |
| TaERF3-2D F |  | ATGAACAAGGTGGCGTCCCG |
| TaERF3-2D R |  | TTCAGGTCGAACCCCACGCC |
| TaERF4-2A F |  | CAGGATGCCAGAGCAACTGC |
| TaERF4-2A R |  | TAGAAGAGCCGCAGCTCCGT |
| TaERF5-6B F |  | CCACATCTAGCCACAGCAGCA |
| TaERF5-6B R |  | GGCGCTGCAACCTCGTCTG |
| TaERF6-3D F |  | CTGCAGCAGCCTGTTCCGT |
| TaERF6-3D R |  | CGGAGGCGAGAAGTAGACGAAG |
| TaERF7-4A F |  | TGCTGAGAAGATCGACGGCAGT |
| TaERF7-4A R |  | GTCCTCGGCGAGACAGTCATC |
| TaERF8-4B F |  | GGAGACCGAGCAGCTGACAAA |
| TaERF8-4B R |  | CGTCGGAGTCGGAGTCTGATG |
| TaERF9-1A F |  | AGCAGCTGGGCGATGGCAAT |
| TaERF9-1A R |  | GGGACCCGGACATCGACTTC |
| TaERF10-1A F |  | AGCAGCATCTCCGCGACGAC |
| TaERF10-1A R |  | GCTTCATCAAGTCCACCAGGCC |
| TaERF11-3D F |  | CAATGAGGCGAGGTGAATGCTAC |
| TaERF11-3D R |  | GGGAATTCAATGTTTCGATGCCT |
| TaERF12-1B F |  | GAAAGAGGATGATGACGAGGGTG |
| TaERF12-1B R |  | TCCCAGATGTCGGCGTCTCTA |
| TaERF13-2B F |  | TCGTGAAAGACAATGCGCAAGTG |
| TaERF13-2B R |  | TGCTCGCTCGATAGGTATCGCA |
| TaERF14-6D F |  | TCTCCTCCTCCCTCCTCAAGC |
| TaERF14-6D R |  | TCATGAGAACGATCAGTGGTTGC |
| TaP5CS1 F |  | GACAAGTCCCGTGTTGGTAGAG |
| TaP5CS1 R |  | CGTGCAGCAACAGCCATTT |
| TaERF3 F |  | AGCAATCAGGCAAAGCAACC |
| TaERF3 R |  | ACGACTCAGAAGGAACCACGAC |
| TaDREB1 F |  | CTCCATTGCCGATGTCTA |
| TaDREB1 R |  | GGATACTTCCAGGGTCTTG |
| TaSOD (Fe) F |  | GGTTGGGTTTGGCTTGTC |
| TaSOD (Fe) R |  | TCGCCTGTCATCCTTGTAAT |
| TaPOD F |  | TTGTGGTGGCGGTGGTAGTGG |
| TaPOD R |  | CGAAGCAGTCGTGGAAGTGGAG |
| TaCAT F |  | GTGCTGAACCGCAACATCG |
| TaCAT R |  | AGCAGCTTGTCGTCCGAGT |
| TaACTIN7 F |  | CAATCTATGAGGGATACACGC |
| TaACTIN7 R |  | GAACCTCCACTGAGAACAACA |
| AtP5CS1 F |  | TTGTGATCCCAAGAGGAAGC |
| AtP5CS1 R |  | CGCTTTGCCATATCCGTATC |
| AtDREB2A F |  | AGGCCCGAGAGTCAACAAAGTG |
| AtDREB2A R |  | CGTCGAAGAATCCATTACCATCC |
| AtRD29A F |  | ACGTTTGCTCCAAGTGGTGA |
| AtRD29A R |  | CCTCCAACGTTATCGGGGTC |
| AtCOR15A F |  | GGTGAGAAAGCGAAAGACTACGT |
| AtCOR15A R |  | CCTCTCCTGCTTTACCCTCCG |
| AtMYB15 F |  | TTTGGTGCGGATATCGATGAAAG |
| AtMYB15 R |  | ACCAGAAGTCCATCTCACTGTC |
| AtERD10 F |  | GAGAAGCTTCCAGGCCACAG |
| AtERD10 R |  | GGGATGTCAGCAATCGGTGTT |
| AtLTI30 F |  | ACTGGGACTAACACGGCTTATGG |
| AtLTI30 R |  | GGTGAACAACGCCAGTATTACCA |
| AtKIN1 F |  | AGCTGAGGAGAAGAGCAATGTT |
| AtKIN1 R |  | CTGCCGCATCCGATACACTCT |
| AtGSTU19 F |  | GTGTTTGCAGAGGGAGAGCGT |
| AtGSTU19 R |  | GCTCAGAGACGAATTCAGTAACC |
| ACTIN2 F |  | CTAAGCTCTCAAGATCAAAGGC |
| ACTIN2 R |  | AACATTGCAAAGAGTTTCAAGG |
| TaERF13-2B F | Full-length ORF cloning | AGACAACAGAGCACGGCGAG |
| TaERF13-2B R |  | CTTGCGCATTGTCTTTCACGATC |
| TaERF13-2B F | Transgenic *Arabidopsis* | acgggggactctagaggatccATGCTGCTGAACCCGGCCTCG |
| TaERF13-2B R |  | cgatcggggaaattcgagctcCTAGCTGACCAGCTGCTGCAC |
| TaERF13-2B F | Transgenic wheat | aggtcgactctagaggatccATGCTGCTGAACCCGGCCTCG |
| TaERF13-2B R |  | tcgagctctctagactagtCTAGCTGACCAGCTGCTGCAC |
| BD-TaERF13-2B F | Transcriptional activity/Yeast two-hybrid assay | atggccatggaggccgaattcATGCTGCTGAACCCGGCCTCG |
| BD-TaERF13-2B R |  | atgcggccgctgcaggtcgacCTAGCTGACCAGCTGCTGCAC |
| BD-TaERF13-2B-N F |  | atggccatggaggccgaattcATGCTGCTGAACCCGGCCTCG |
| BD-TaERF13-2B-N R |  | atgcggccgctgcaggtcgacCTTCCCCCTCGCCACGGCCG |
| BD-TaERF13-2B-C F |  | atggccatggaggccgaattcATGCACTACCGCGGGGTGAGGCA |
| BD-TaERF13-2B-C R |  | atgcggccgctgcaggtcgacCTAGCTGACCAGCTGCTGCAC |
| AD-TaVDAC F |  | gccatggaggccagtgaattcATGGGCGGCCCAGGCCTCTA |
| AD-TaVDAC R |  | cagctcgagctcgatggatccTCAAGGCTTGAGAGCAATAGC |
| AD-TaNRX F |  | gccatggaggccagtgaattcATGGCGGCGTCCCCCACCCCC |
| AD-TaNRX R |  | cagctcgagctcgatggatccCTAGGCCTTCCTGCAGACGTC |
| AD-TaG6PE F |  | gccatggaggccagtgaattcATGGCGGCTTCCTGCACGCTC |
| AD-TaG6PE R |  | cagctcgagctcgatggatccTCAGACAAGTTCAATTTTCTG |
| AD-TaCIPK9 F |  | gccatggaggccagtgaattcATGGCGGCGGCGGGCGGGGG |
| AD-TaCIPK9 R |  | cagctcgagctcgatggatccCTATTTCGCAATTGTGTTGGA |
| AD-TaCab F |  | gccatggaggccagtgaattcATGGCGGCCACCACCATGTC |
| AD-TaCab R |  | cagctcgagctcgatggatccTTACTTCCCGGGCACGAAGT |

| Table S2. Physical locations and properties of ERF members in wheat | | | | | | |
| --- | --- | --- | --- | --- | --- | --- |
| Name | Gene ID | Class | Protein size | Molecular weight (KDa) | Theoretical pI | Subcellular location |
| TaERF1-1A | TraesCS1A02G314300.1 | I | 236 | 24220 | 8.87 | Nucleus |
| TaERF1-1B | TraesCS1B02G326600.1 | I | 240 | 24430.19 | 8.87 | Nucleus |
| TaERF1-1D | TraesCS1D02G314800.1 | I | 236 | 24242.09 | 8.87 | Nucleus |
| TaERF2-3A | TraesCS3A02G328000.1 | I | 247 | 25057.07 | 9.01 | Nucleus |
| TaERF2-3B | TraesCS3B02G357500.1 | I | 243 | 24751.65 | 9.24 | Nucleus |
| TaERF2-3D | TraesCS3D02G321500.1 | I | 246 | 24977.94 | 9.24 | Nucleus |
| TaERF3-2A | TraesCS2A02G427700.1 | II | 210 | 21441.45 | 9.2 | Nucleus |
| TaERF3-2B | TraesCS2B02G448100.1 | II | 219 | 22278.39 | 7.9 | Nucleus |
| TaERF3-2D | TraesCS2D02G425700.1 | II | 217 | 22065.25 | 9.2 | Nucleus |
| TaERF4-2A | TraesCS2A02G542700.1 | III | 193 | 20031.67 | 9.19 | Nucleus |
| TaERF4-2B | TraesCS2B02G572500.1 | III | 191 | 19957.46 | 9.19 | Nucleus |
| TaERF4-2D | TraesCS2D02G543900.1 | III | 193 | 20057.49 | 8.72 | Nucleus |
| TaERF5-6B | TraesCS6B02G159600.1 | III | 203 | 20684.04 | 6.6 | Nucleus |
| TaERF5-6D | TraesCS6D02G121100.1 | III | 204 | 20766.1 | 6.6 | Nucleus |
| TaERF6-3A | TraesCS3A02G328100.1 | IV | 173 | 18116.48 | 9.89 | Nucleus |
| TaERF6-3B | TraesCS3B02G357600.1 | IV | 174 | 18425.77 | 9.88 | Nucleus |
| TaERF6-3D | TraesCS3D02G321700.1 | IV | 173 | 18343.71 | 10.07 | Nucleus |
| TaERF7-4A | TraesCS4A02G005500.1 | V | 296 | 31791.97 | 4.45 | Nucleus |
| TaERF7-4B | TraesCS4B02G299600.1 | V | 293 | 31586.94 | 4.74 | Nucleus |
| TaERF8-4A | TraesCS4A02G005400.1 | V | 330 | 35081.7 | 4.56 | Nucleus |
| TaERF8-4B | TraesCS4B02G299700.1 | V | 336 | 35956.59 | 4.67 | Nucleus |
| TaERF8-4D | TraesCS4D02G298600.1 | V | 342 | 36512.05 | 4.6 | Nucleus |
| TaERF9-1A | TraesCS1A02G370700.1 | VI | 265 | 28656.93 | 5.99 | Nucleus |
| TaERF10-1A | TraesCS1A02G370600.1 | VI | 256 | 27792.03 | 5.69 | Nucleus |
| TaERF10-1D-1 | TraesCS1D02G376600.1 | VI | 265 | 28969.37 | 4.98 | Nucleus |
| TaERF10-1D-2 | TraesCS1D02G376700.1 | VI | 266 | 28747.13 | 6 | Nucleus |
| TaERF11-3D | TraesCS3D02G291100.1 | VII | 181 | 18476.8 | 9.64 | Nucleus |
| TaERF12-1A | TraesCS1A02G218100.1 | VII | 236 | 26381.65 | 5.5 | Nucleus |
| TaERF12-1B | TraesCS1B02G231500.1 | VII | 256 | 28052.48 | 6 | Nucleus |
| TaERF13-2A | TraesCS2A02G417300.1 | VIII | 298 | 31376.09 | 5.68 | Nucleus |
| TaERF13-2B | TraesCS2B02G436300.1 | VIII | 290 | 30666.35 | 5.68 | Nucleus |
| TaERF14-6A | TraesCS6A02G243300.1 | VIII | 272 | 28478.07 | 5.96 | Nucleus |
| TaERF14-6D | TraesCS6D02G225500.1 | VIII | 273 | 28705.36 | 5.97 | Nucleus |

| Table S3. *Arabidopsis* and *Brachypodium* members used | |
| --- | --- |
| Name | Gene ID |
| BdERF1 | BRADI_2g21060v3 |
| BdERF2 | BRADI_2g52370v3 |
| BdERF3 | BRADI_5g21250v3 |
| BdERF4 | BRADI_5g25570v3 |
| BdERF5 | BRADI_3g04410v3 |
| BdERF6 | BRADI_2g52380v3 |
| BdERF7 | BRADI_1g72449v3 |
| BdERF8 | BRADI_2g50035v3 |
| BdERF9 | BRADI_2g16442v3 |
| BdERF10 | BRADI_5g17480v3 |
| BdERF11 | BRADI_3g50490v3 |
| AtERF1 | AT3G20310 |
| AtERF2 | AT3G14230 |
| AtERF3 | AT2G44840 |
| AtERF4 | AT5G47220 |

| Table S4. The putative TaERF13-2B interaction partners in wheat | | | |
| --- | --- | --- | --- |
| Gene | Locus ID | Putative protein | Accession |
| *TaVDAC* | LOC123111779 | Mitochondrial outer membrane porin | XM_044532654.1 |
| *TaNRX* | LOC123190474 | Nucleoredoxin | XM_044603121.1 |
| *TaG6PE* | LOC123121283 | Glucose-6-phosphate-1-epimerase | XM_044541187.1 |
| *TaCIPK9* | LOC123093554 | CBL-interacting protein kinase 9 | XM_044515537.1 |
| *TaCAB* | LOC123183162 | Chlorophyll a/b binding protein | XM_044595913.1 |
